# Oropouche Virus Importation in Southern Brazil and Emerging Concern Calling for Enhanced Public Health Surveillance

**DOI:** 10.1002/jmv.70557

**Published:** 2025-08-11

**Authors:** Franciellen Machado dos Santos, Elverson Soares de Melo, Gustavo Barbosa de Lima, Alexandre Freitas da Silva, Marcelo Henrique Santos Paiva, Bartolomeu Acioli‐Santos, Clarice Neuenschwander Lins de Moraes, Amanda Pellenz Ruivo, Tatiana Schäffer Gregianini, Milena Bauermann, Thales Bermann, Valeska Lizzi Lagranha, Ludmila Fiorenzano Baethgen, Fernanda Godinho, Gabriel da Luz Wallau, Ana Beatriz Gorini da Veiga, Richard Steiner Salvato

**Affiliations:** ^1^ Programa de Pós‐graduação em Biociências Universidade Federal de Ciências da Saúde de Porto Alegre Porto Alegre Brazil; ^2^ Departamento de Entomologia Instituto Aggeu Magalhães, Fundação Oswaldo Cruz Recife Brazil; ^3^ Departamento de Virologia Instituto Aggeu Magalhães, Fundação Oswaldo Cruz Recife Brazil; ^4^ Centro Estadual de Vigilância em Saúde, Secretaria Estadual de Saúde do Rio Grande do Sul Porto Alegre Brazil; ^5^ Universidade Luterana do Brasil Canoas Brazil; ^6^ Universidade Federal do Rio Grande do Sul Porto Alegre Brazil; ^7^ Núcleo de Bioinformática, Instituto Aggeu Magalhães, Fundação Oswaldo Cruz Recife Brazil; ^8^ Department of Arbovirology and Entomology Bernhard Nocht Institute for Tropical Medicine Hamburg Germany; ^9^ Universidade Federal de Santa Maria Santa Maria Rio Grande do Sul Brazil

**Keywords:** arbovirus, Brazil, Oropouche, surveillance, virus emergence

## Abstract

Oropouche virus (OROV), an arthropod‐borne virus transmitted by *Culicoides paraensis*, is an endemic arbovirus that historically circulates mostly in the Amazon basin. Between 2022 and 2024, it reemerged as a more widespread public health concern in South America. We conducted a pooled‐sample molecular surveillance study to understand the prevalence of Oropouche fever in Brazil's southernmost state. Over 18 months, we analyzed 4060 samples to monitor the virus emergence in the Rio Grande do Sul state. We detected the first human case of OROV in the state, and our phylogenetic reconstruction indicated a travel‐related introduction from the Amazon region into Rio Grande do Sul. Despite the absence of local transmission, the invasion of *Culicoides paraensis* and enzootic circulation of the OROV in Rio Grande do Sul highlight the risk of Oropouche fever outbreaks in the region. We demonstrated that pooled‐sample surveillance effectively monitors virus introduction during periods of low endemic circulation, serving as an essential active surveillance tool for the timely detection of virus emergence and enhancing public health preparedness. The multiple introductions of distinct OROV lineages into southern Brazil underscore the importance of genomic surveillance and public health strategies to monitor and mitigate arbovirus spread in the region.

## 1 Introduction

Oropouche virus (OROV) is a reemerging arthropod‐borne virus (arbovirus) primarily transmitted by *Culicoides paraensis*. The virus is maintained in a zoonotic transmission cycle, including vertebrate hosts such as pale‐throated sloths, rodents, nonhuman primates, and other wild mammals [1]. Oropouche fever is usually characterized by mild symptoms, starting with the abrupt onset of fever and accompanied by severe headache, chills, myalgia, arthralgia, photophobia, dizziness, retro‐orbital pain, nausea, and vomiting. However, in some cases, the disease can progress to more severe manifestations, including hemorrhagic symptoms, meningitis, and meningoencephalitis [2, 3]. Recently, complications during pregnancy have been linked to OROV infection, with cases of vertical transmission reported in Brazil [4, 5]. These have been associated with adverse pregnancy outcomes, including fetal death and congenital abnormalities [6]. Neither vaccines nor antiviral drugs are available to prevent OROV infection or treat individuals affected by Oropouche fever [2].

The first OROV infections have been documented in the Amazon basin since the 1950s [1]. However, as a typical neglected tropical disease, its real burden has remained largely unknown until recently. Current estimates indicate that more than half a million human cases may have occurred since the virus was first identified [5]. In recent years (2022–2025), a significant increase in Oropouche fever cases has been observed in Brazil and other countries such as Bolivia, Colombia, Peru, and Cuba [7]. In Brazil, outbreaks have primarily been reported in the northern states, though different regions outside the endemic areas have also been affected. By April 2025, over 23 000 laboratory‐confirmed cases had been reported in Brazil, with almost the totality of the cases reported in the current year occurring in regions considered non‐endemic for virus circulation [5]. A genomic study identified that these outbreaks were caused by a new reassortant virus, which contains genomic segments from different viruses classified within the species *Orthobunyavirus oropoucheense* [8]. As of April 2025, OROV local transmission has been identified in 23 of the 27 Brazilian states, except for Rio Grande do Norte, Goiás, Paraná, and Rio Grande do Sul, which have only reported imported cases. To date, four deaths associated with the virus have been confirmed, along with five cases of vertical transmission, including four fetal deaths and one congenital anomaly [5, 9].

Accurately estimating the true prevalence of OROV infections remains challenging due to significant surveillance biases. Most existing data come from passive surveillance during outbreaks, which primarily captures symptomatic cases while underrepresenting asymptomatic infections. The absence of data on asymptomatic and afebrile individuals limits our understanding of important disease characteristics, such as the proportion of OROV infections that present with clinical symptoms [10]. Additionally, OROV infection presents with clinical manifestations that closely mirror those of other arboviruses such as dengue, Zika and chikungunya, creating significant diagnostic challenges in regions where these viruses co‐circulate [11]. Molecular diagnosis is currently the primary tool for detecting arboviral infections and estimating disease incidence and prevalence [5]. These methods require sample collection during the viremic window, which typically lasts up to 5 days after symptom onset [12]. In endemic areas, the concurrent circulation of different arboviruses can overwhelm laboratory capacities, further impeding timely diagnosis. To address this issue, alternative approaches such as pooled RT‐PCR testing can be implemented. This strategy can significantly increase testing throughput while maintaining diagnostic sensitivity, particularly during outbreaks [13].

Rio Grande do Sul, the southernmost state in Brazil, has historically been less affected by arboviruses compared to other regions of the country, with dengue and chikungunya infections mostly associated with travel‐associated introductions resulting in self‐limited transmission [14]. However, since 2021, the state has experienced a significant upsurge in dengue infections, marked by widespread viral expansion and large annual outbreaks. Rio Grande do Sul benefits from a well‐established serological and molecular surveillance infrastructure for monitoring arboviruses such as dengue, Zika, and chikungunya, coordinated by its central public health laboratory [15, 16]. But, despite the importance of OROV emergence and its rapid expansion across Brazil, routine diagnostic testing for this virus has not been widely and systematically implemented in arbovirus surveillance conducted by public health laboratories, including in Rio Grande do Sul.

In light of the emergence of OROV cases in the country, we conducted an active surveillance study to detect the virus in samples from individuals who tested negative for other arboviruses (dengue, Zika, and chikungunya) through routine surveillance performed by the Rio Grande do Sul Public Health Laboratory. We aimed to better understand the prevalence of Oropouche fever in the southernmost state of Brazil, to guide timely public health interventions.

## 2 Methods

### 2.1 Population

From January 2023 to June 2024, Rio Grande do Sul accounted for 245 000 cases of arbovirus infections, almost the totality of these cases due to dengue virus infection. In the study period, the Rio Grande do Sul Public Health Laboratory (LACEN‐RS) received 62 700 serum samples from cases suspected of infection by arboviruses. These samples were analyzed using antigen tests to detect the dengue virus's nonstructural protein 1 (NS1), immunoglobulin (Ig)M antibodies and RT‐PCR for dengue, Zika, and chikungunya ribonucleic acid (RNA) detection. Of the samples, 37 700 were collected within the first 5 days of symptom onset. We then performed a probabilistic random selection, considering the collection date and city, to choose 4060 samples collected within 5 days of symptom onset, with negative results for the tested viruses (dengue, Zika, and chikungunya) in routine surveillance.

### 2.2 Pooled‐Sample Oropouche Virus Detection

The 4060 serum samples were pooled into 406 pools, with 10 samples per pool. The chosen pool size was based on previous evidence demonstrating that this pool size provides an optimal balance between diagnostic sensitivity and cost‐effectiveness [17, 18], while also allowing for efficient grouping of samples by shared temporal and geographic characteristics. These pools were formed by grouping samples from the same geographic region (e.g., city) and with similar collection times to achieve geographic and temporal closeness.

For pooling the samples, 50 µL of serum from each of the 10 samples in the same pool were combined in a microtube, resulting in a total volume of 500 µL. Subsequently, 200 µL of this volume was used for nucleic acid extraction. The extraction was performed on Extracta‐96 equipment (Loccus, São Paulo, Brazil) using the Fast Kit–Viral DNA and RNA (MVXA‐P096 FAST), according to the manufacturer's instructions. The OROV detection was performed through reverse transcription‐quantitative polymerase chain reaction (RT‐qPCR) using a previously published protocol [19]. We used SuperScript III Platinum One‐Step qRT‐PCR Kit (Thermo Fisher Scientific), following the manufacturer's recommended cycling conditions: 50°C for 15 min for reverse transcription, 95°C for 2 min for initial denaturation/enzyme activation, followed by 45 cycles of 95°C for 15 s and 60°C for 30 s. Amplification reactions were performed in a CFX Opus Real‐Time PCR System (Bio‐Rad, Hercules, CA, USA). Viral detection was considered positive for cycle threshold (Ct) values ≤ 40.

In cases where a pooled sample tested positive, each sample within the pool was subsequently tested separately to identify the positive case. This process involved re‐extracting RNA from the original individual samples and performing RT‐qPCR under the same conditions used for pooled testing.

### 2.3 Viral Sequencing and Genome Assembly

OROV genome amplification and sequencing library was prepared using the Illumina COVIDSeq Kit (Illumina, San Diego, USA), replacing the SARS‐CoV‐2 primers with previously designed OROV primers [8]. Genome sequencing was conducted on the MiSeq platform with the V2 reagent kit, employing a paired‐end approach with 2 × 150 cycles. The resulting sequencing reads were processed using the ViralFlow pipeline (version 1.2.0) [20], applying a reference‐guided genome assembly strategy. Each of the three genomic segments of OROV—L (OL689334.1), M (OL689333.1), and S (OL689332.1) was assembled independently using the corresponding reference sequences.

### 2.4 Phylogenetic Analysis

We conducted a genomic alignment and phylogenetic reconstruction to place the OROV sample collected in Rio Grande do Sul within the phylogenetic context of the virus spread across Brazil. The sequenced genome was compared with representative OROV genomes to elucidate phylogenetic relationships, transmission dynamics, and potential epidemiological implications. We included OROV genomes from Gräf et al. [21] and Naveca et al. [8], as well as additional genomic sequences from samples available in the GISAID database (Global Data Science Initiative) [22], through the EpiArbo database, retrieved up to January 10, 2025. Concatenated OROV segments were aligned using MAFFT (version 7.490) [23], followed by manual curation to remove unaligned regions and noncoding sequences.

The alignment of concatenated segments was analyzed using ModelFinder [24] to decide the most appropriate nucleotide substitution model. The sequences were then processed in BEAST (version 1.10.4) [25] to reconstruct a Bayesian time‐scaled phylogenetic tree. Sample collection dates were incorporated as Tip Dates. The analysis was conducted under the GTR + G + I substitution model, with a partitioning scheme that treated each codon position separately. An uncorrelated lognormal relaxed clock model was also applied. As a tree prior, the coalescent Bayesian Skyline model was used, enabling the inference of changes in adequate population size over time without imposing strict parametric assumptions on viral demography. Ten independent runs were performed using the Markov Chain Monte Carlo (MCMC) for the phylogenetic inference method, each consisting of 100 million generations. The chains from all runs were combined using LogCombiner (part of the BEAST package), discarding the first 10% of states as burn‐in. Chain convergence was assessed by calculating the Effective Sample Size (ESS) for all parameters using Tracer (version 1.7.1) [26], ensuring values above 200 for a robust estimation of posterior distributions. The consensus phylogenetic tree was obtained using the maximum clade credibility criterion with TreeAnnotator (part of the BEAST package) and visualized and annotated in FigTree.

## 3 Results

**Figure 1:**
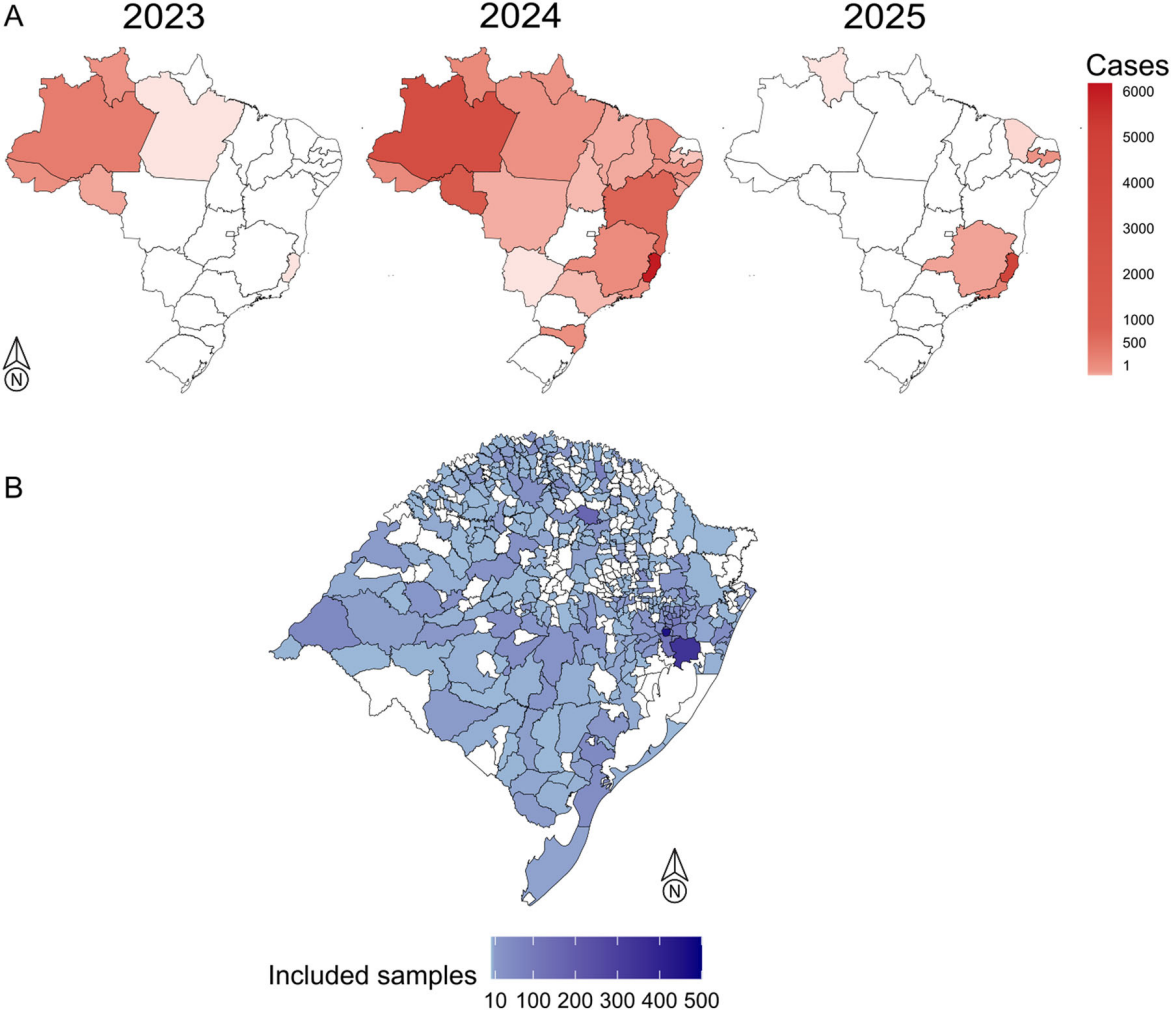
Distributions of confirmed Oropouche fever cases reported in Brazil from 2023 to 2025 and geographic distribution of the samples included in this study. (A) Number of confirmed Oropouche fever cases reported in Brazilian states per year, including data up to February 18, 2025, as reported by the Brazilian Ministry of Health. (B) The map of Rio Grande do Sul state, showing the geographic distribution of clinical samples from individuals presenting arbovirus infection symptoms, tested for OROV infection in this study.

In 2023, Brazil reported a total of 832 cases of Oropouche fever, with the majority of these cases concentrated in the endemic Amazon Basin region. In 2024, the number of Oropouche fever cases surged to 13 783, expanding across all Brazilian regions. By 2025, the pattern persisted, with 4857 cases reported so far, the majority of which occurred outside the historically endemic Amazon Basin region, highlighting a significant shift in the epidemiology of the disease (Figure 1A).

Among the 4060 samples tested (Figure 1B), a single positive case for OROV was identified, showing Ct values of 17 in pooled‐sample testing and 16 upon individual retesting. The sample was obtained from an individual diagnosed in Erechim, located in the northern region of Rio Grande do Sul state. The patient, a resident of the bordering municipality of Aratiba, presented with symptoms including fever, myalgia, and a rash on January 8, 2024. The NS1 test to detect the nonstructural protein 1 of the dengue virus was negative, while RT‐PCR confirmed the presence of OROV. The case was considered likely imported, as the individual had traveled to the northern region of Brazil 1 week before the symptoms onset.

**Figure 2:**
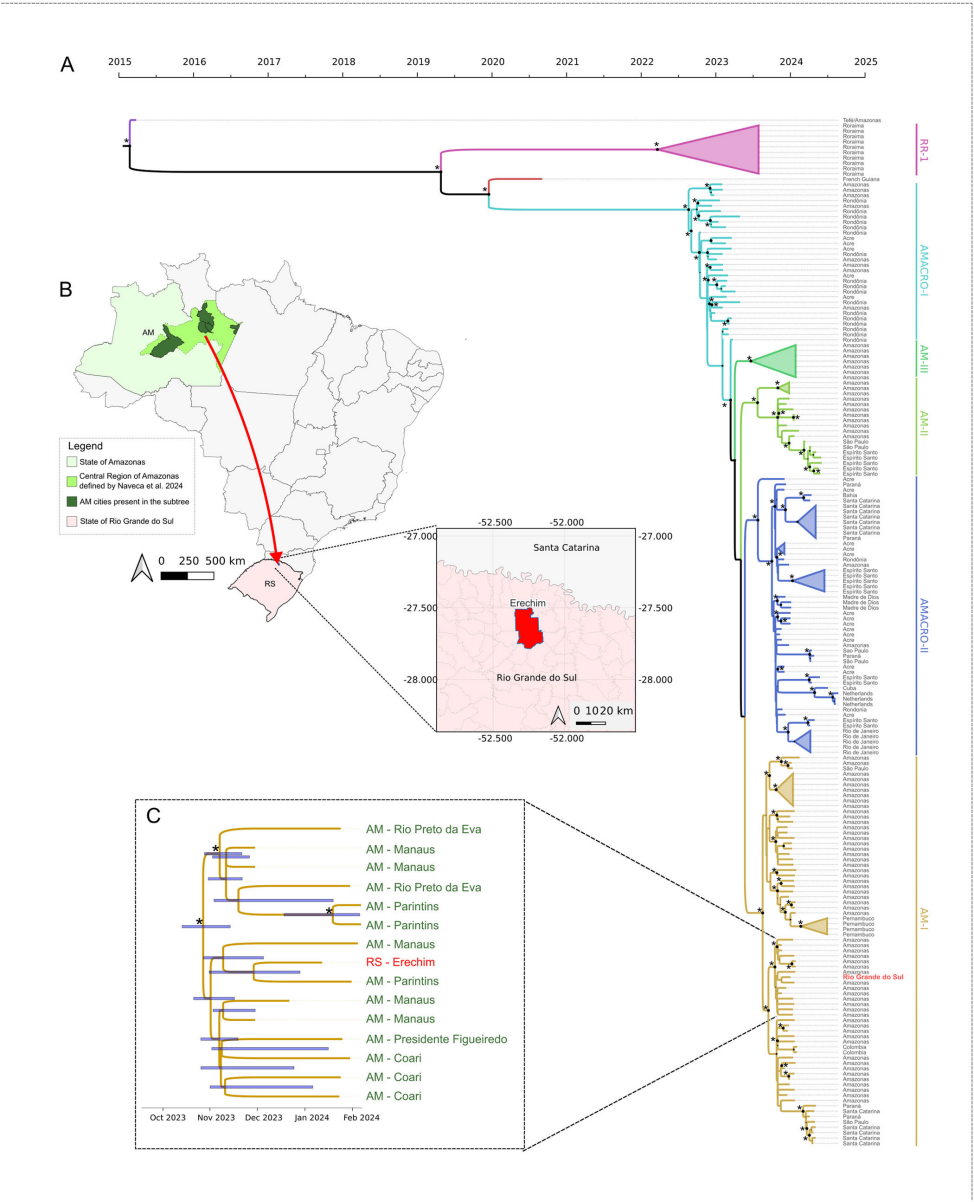
Phylogenetic analysis of Oropouche virus (OROV) in Brazil, highlighting a sample from Rio Grande do Sul. (A) Maximum Clade Credibility (MCC) time‐scaled phylogenetic tree showing six major OROV clades defined by Naveca et al. (2024). The Rio Grande do Sul sample (red) clusters within the AM‐I lineage from Amazonas. A subtree within this well‐supported clade (posterior probability = 1) includes sequences from Erechim (Rio Grande do Sul) alongside five Amazonas cities. The Most Recent Common Ancestor (MRCA) is estimated to have emerged between mid‐October and mid‐November 2023. The map in (B) shows the transmission route from the central region of Amazonas to Erechim. (C) Zoomed‐in view of the clade containing the Erechim (Rio Grande do Sul) sequence. The asterisks in the left side of nodes in (A) and (C) represent posterior probabilities higher than 0.9. In (A), these probabilities are visually represented by gradually sized points at the tree's nodes.

The reconstructed time‐scaled phylogeny of the virus's evolution in Brazil (Figure 2A) indicates that the OROV sample isolated in Erechim clusters with sequences from the state of Amazonas within the AM‐I clade. This clade comprises isolates from the central region of Amazonas, where the virus circulated actively between November 2023 and February 2024, coinciding with the reported travel period of the patient to the region. In addition to this travel‐related viral introduction into Rio Grande do Sul, we identified that available genomes from Paraná and Santa Catarina, two other southern Brazilian states, did not cluster with the genome from the patient diagnosed in Rio Grande do Sul, further supporting the travel‐related infection. This data reinforces that the case analyzed in this study did not result from a viral importation from the neighboring states.

A more detailed analysis of the highly supported subclade where the Erechim sample is positioned (Figure 2C), indicates that this virus shares a recent common ancestor with samples from central Amazonas (highlighted in light green in Figure 2B), including ones from Rio Preto da Eva, Parintins, Presidente Figueiredo, Coari, and Manaus cities. The estimated time to the most recent common ancestor (TMRCA) suggests that this subclade emerged between mid‐October and mid‐November 2023, coinciding with the peak circulation of the AM‐I clade in the central region of Amazonas, occurring just 1 month before the travel of the positive Erechim individual to Manaus, Amazonas. These findings, in addition to the travel history of this individual diagnosed with Oropouche fever, support the hypothesis that the infection was acquired in the Central Region of Amazonas and subsequently introduced directly into Erechim, as illustrated in Figure 2B.

## 4 Discussion

During the study period, Brazil faced a significant OROV outbreak, marked by the virus's spread from the Amazon Basin region to other parts of the country. This was accompanied by a notable shift in the epidemiology of Oropouche fever, with increasing cases now emerging in the Southeast of Brazil. As of February 2025, 23 out of Brazil's 27 states had reported cases of Oropouche fever, highlighting the expanding spread of the virus across the country [9]. Notably, Rio Grande do Sul, the southernmost Brazilian state, did not register any cases during this period. We employed pooled‐sample testing to enhance surveillance and investigate the potential introduction of the OROV in the region. Out of the 4060 samples tested, we identified only one positive sample for OROV, demonstrating the ability of pooled surveillance testing to detect infections even in scenarios of low viral circulation.

Pooled‐sample surveillance has emerged as a highly effective strategy for enhancing the efficiency of clinical diagnostics [27]. By significantly reducing the time, labor, and reagents required for large‐scale testing, this method enables efficient screening for pathogens in large asymptomatic populations. Its simplicity, alignment with existing approved procedures, and lack of need for specialized sample handling or additional information make it an easily scalable solution [28]. These advantages establish pooled‐sample surveillance as a practical and adaptable tool for public health screening, particularly in scenarios demanding rapid and widespread testing, such as epidemics or outbreaks [29, 30]. However, most reported implementations of this approach focus on SARS‐CoV‐2, and there is a lack of evidence demonstrating the use of pooled surveillance testing for arbovirus detection [31]. In this context, the ongoing large‐scale dengue epidemics in Brazil have significantly strained laboratory capacity, limiting the feasibility of implementing new diagnostics for other emerging viruses [32]. Given these constraints, pooled‐sample surveillance emerges as a valuable complementary strategy to enhance pathogen detection at the national level. This approach can optimize resource use and broaden the reach of surveillance efforts, particularly for the early detection of emerging or re‐emerging pathogens that could remain undetected in the current surveillance system [17]. Despite the lack of studies evaluating pooled‐sample surveillance for arboviruses, and considering the transient nature of OROV viremia with typically low RNA levels in serum or plasma, our results suggest that pooling can still be a useful screening approach, particularly in resource‐limited settings or during periods of high testing demand.

It is also important to note that the sensitivity of an arbovirus surveillance system depends on several factors, including the knowledge and awareness of local clinicians and healthcare services about potential arboviruses circulating, or at risk of being introduced, in the region, as well as the need to request testing for these pathogens [33]. Furthermore, since many arbovirus infections present as mild or even asymptomatic cases, affected individuals often do not seek healthcare services, which further limits detection and allows for potential undetected viral circulation in the population [34]. Therefore, implementing and maintaining routine passive surveillance for high‐risk arboviruses at the regional level is essential for detecting emerging viral threats. This can be enhanced through pooled‐sample testing strategies, which can improve surveillance efficiency and effectiveness. Studies such as this one highlight and reinforce the importance of differential diagnosis in arboviral surveillance. Moreover, our findings can serve as a tool to raise awareness among healthcare professionals about the critical role of sample collection in improving the sensitivity and reach of arbovirus surveillance systems.

The time‐scaled phylogeny of the detected OROV revealed critical patterns of viral spread, particularly between northern and southern regions. The sample detected in Erechim, Rio Grande do Sul, clustered within the AM‐I clade, linking it to active viral circulation in central Amazonas between late 2023 and early 2024. The estimated emergence of this subclade in mid‐October to mid‐November 2023, coupled with the patient's travel history to Manaus, supports the hypothesis of direct viral introduction from Amazonas to Rio Grande do Sul. Furthermore, distinct introductions into neighboring Santa Catarina and Paraná states, represented in separate clades, further support the hypothesis of viral importation from the Amazon region rather than local transmission across bordering states. These findings emphasize the crucial role of genomic surveillance in monitoring arbovirus spread, especially in areas with high human mobility and ecological diversity. Identifying multiple independent introductions into southern Brazil stresses the need for enhanced public health strategies to monitor and mitigate the spread of emerging arboviruses [22].

In early 2024, an infestations of *Culicoides paraensis*, the main OROV vector, were detected in seven municipalities from Rio Grande do Sul state (Mampituba, Três Forquilhas, Dom Pedro de Alcântara, Itati, Maquiné, Mampituba, and Terra de Areia). These findings were generated from entomological surveillance activities conducted by the State Center for Health Surveillance and the Department of Agriculture, Livestock, Sustainable Production, and Irrigation of Rio Grande do Sul [35] revealing a concerning scenario for the potential transmission of the virus, as the presence of *Culicoides paraensis* in the region creates an environment conducive to the spread of the virus. Aside from the invasion of the biting midge, previous studies conducted in the area have also reported the enzootic circulation of OROV, evidenced by antibodies in nonhuman primates in 2004, 2012, and 2014 [36]. This combination of vector presence and historical evidence of viral circulation increases the risk of Oropouche fever outbreaks in the region, given the potential for both vector expansion and ongoing enzootic circulation.

In recent years, Rio Grande do Sul has experienced a change in dengue occurrence patterns, from self‐limited transmission due to virus importation events to annual large dengue virus outbreaks by lineages that are now endemic in the region. This change in the dengue transmission pattern led to a significant increase in the burden of dengue‐related morbidity and mortality, as the Rio Grande do Sul had a naive population for dengue infection [37]. This epidemiological change is mainly associated with *Aedes aegypti* (the primary dengue mosquito vector in Brazil), which spread to southern areas due to the erosion of the climatic barrier related to climate change, leading to new outbreaks in an immunologically naive human population [16]. A similar scenario is being remounted in Rio Grande do Sul, where an entirely naive population for OROV is present. As evidenced by entomological surveillance data, the region has witnessed an invasion of *Culicoides paraensis*. This complex scenario of rapid viral expansion, spread of *Culicoides paraensis*, and the current lack of Oropouche surveillance in Southern Brazil presents a significant public health threat, leaving the region highly vulnerable to potential outbreaks.

The occurrence of a potential OROV outbreak in Southern Brazil, along with the increasing annual outbreaks of dengue virus occurring in recent years, could dangerously increase the arboviral disease burden in the region, leading to overcrowding the healthcare services, increased demand for resources such as medical supplies and healthcare professionals, resulting in significant social and economic impact. Active surveillance allows the early detection of viral introduction and spread, enabling the timely implementation of disease control measures such as vector control, health education campaigns, and healthcare system preparedness. This proactive approach is crucial in preventing a health crisis and averting the collapse of medical services [38].

Our study demonstrated that during periods of low endemic circulation, sentinel laboratory surveillance using a pooled sampling approach can serve as an effective alternative for monitoring the potential introduction of new pathogens. This method proves valuable in settings already burdened with other diseases, such as dengue, by providing efficient surveillance without overwhelming existing routines.

## Author Contributions

**Franciellen Machado dos Santos:** conceptualization, investigation, methodology, data curation, formal analysis, writing – original draft, visualization, writing – review and editing. **Elverson Soares de Melo, Gustavo Barbosa de Lima, Alexandre Freitas da Silva, Amanda Pellenz Ruivo, Tatiana Schäffer Gregianini, Milena Bauermann, Thales Bermann, Valeska Lizzi Lagranha, Ludmila Fiorenzano Baethgen, and Fernanda Godinho:** investigation, methodology, data curation, formal analysis, writing – original draft, visualization, writing – review and editing. **Marcelo Henrique Santos Paiva, Bartolomeu Acioli‐Santos, Clarice Neuenschwander Lins de Moraes:** formal analysis, writing – review and editing. **Gabriel da Luz Wallau:** funding acquisition, formal analysis, writing – review and editing. **Ana Beatriz Gorini da Veiga:** supervision, writing – review and editing. **Richard Steiner Salvato:** conceptualization, project administration, funding acquisition, supervision, resources, writing – review and editing.

## Ethics Statement

This project was approved by the Research Ethics Committee (CEP) at Escola de Saúde Pública (SES‐RS). Process number: CAAE: 67181123.1.0000.5312.

## Conflicts of Interest

The authors declare no conflicts of interest.

## Supporting information


**Supplementary Table 1** ‐ GISAID acknowledgement table including sequences included in the phylogenetic analysis.

## Data Availability

The near‐complete genome sequence generated in this study has been deposited in the GISAID Database under accession number EPI_ISL_19742562.
