# Supplementary material for: Oropouche Virus Importation in Southern Brazil and Emerging Concern Calling for Enhanced Public Health Surveillance
Source: J Med Virol. 2025 Aug 11;97(8):e70557. doi: 10.1002/jmv.70557 (PMC12338014; doi:10.1002/jmv.70557)
Supplement: Supplementary file 1 — Supplementary Table 1 ‐ GISAID acknowledgement table including sequences included in the phylogenetic analysis. [file JMV-97-e70557-s001.pdf]

SUPPLEMENTAL TABLE

Data Availability

GISAID Identifier:	EPI_SET_250604ry
DOI:	https://doi.org/10.55876/gis8.250604ry

All genome sequences and associated metadata in this dataset are published in GISAID's EpiArbo database. To view the contributors of each individual sequence with details such as accession number, Virus name, Collection date, Originating Lab and Submitting Lab and the list of Authors, visit 10.55876/gis8.250107tk

Data Snapshot

EPI_SET_250604ry is composed of 17 individual genome sequences.
The collection dates range from 2023-03-21 to 2024-09-02;
Data were collected in 3 countries and territories.
